# Semen molecular and cellular features: these parameters can reliably predict subsequent ART outcome in a goat model

**DOI:** 10.1186/1477-7827-7-125

**Published:** 2009-11-09

**Authors:** Fiammetta Berlinguer, Manuela Madeddu, Valeria Pasciu, Sara Succu, Antonio Spezzigu, Valentina Satta, Paolo Mereu, Giovanni G Leoni, Salvatore Naitana

**Affiliations:** 1 Department of Animal Biology, University of Sassari, Via Vienna 2, 07100 Sassari, Italy; 2 Presidenza, Biblioteca Veterinaria, Faculty of Veterinary Medicine, University of Sassari, Via Vienna 2, 07100 Sassari, Italy; 3 Department of Physiological, Biochemical and Cellular Science, University of Sassari, Via Vienna 2, 07100 Sassari, Italy

## Abstract

Currently, the assessment of sperm function in a raw or processed semen sample is not able to reliably predict sperm ability to withstand freezing and thawing procedures and in vivo fertility and/or assisted reproductive biotechnologies (ART) outcome. The aim of the present study was to investigate which parameters among a battery of analyses could predict subsequent spermatozoa in vitro fertilization ability and hence blastocyst output in a goat model. Ejaculates were obtained by artificial vagina from 3 adult goats (Capra hircus) aged 2 years (A, B and C). In order to assess the predictive value of viability, computer assisted sperm analyzer (CASA) motility parameters and ATP intracellular concentration before and after thawing and of DNA integrity after thawing on subsequent embryo output after an in vitro fertility test, a logistic regression analysis was used. Individual differences in semen parameters were evident for semen viability after thawing and DNA integrity. Results of IVF test showed that spermatozoa collected from A and B lead to higher cleavage rates (0 < 0.01) and blastocysts output (p < 0.05) compared with C. Logistic regression analysis model explained a deviance of 72% (p < 0.0001), directly related with the mean percentage of rapid spermatozoa in fresh semen (p < 0.01), semen viability after thawing (p < 0.01), and with two of the three comet parameters considered, i.e tail DNA percentage and comet length (p < 0.0001). DNA integrity alone had a high predictive value on IVF outcome with frozen/thawed semen (deviance explained: 57%). The model proposed here represents one of the many possible ways to explain differences found in embryo output following IVF with different semen donors and may represent a useful tool to select the most suitable donors for semen cryopreservation.

## Background

During the last decade several molecular and cellular markers have been proposed as tools to evaluate sperm fertility *in vitro *in raw or processed semen samples, but with highly variable results [reviewed in Refs. [1,2]].

Energy metabolism is a key factor supporting sperm function. ATP is one of the basic components in a sperm cell and is used not only as a energy source but also for protein phosphorylation in cell signalling and as a cofactor regulating protein function [3]. The functional integrity of mitochondria is believed to be important for sperm survival in the female genital tract or during assisted reproductive biotechnologies (ART) [4]. In sperm, ATP production supports multiple cellular activities and biochemical events required for successful fertilization to occur, such as capacitation [5,6], acrosome reaction [7] and motility [3]. Recently, sperm oxygen consumption has been correlated with bull fertility and measurement of total ATP formation has been proposed as a test for bull fertilizing ability after freezing and thawing [8]. We reported for vulture spermatozoa that an higher ATP intracellular concentration in fresh semen was followed by a higher survival in vitro after cryopreservation [9]. Furthermore, ATP values correlated positively with sperm viability both before and after cryopreservation [9]. Sperm metabolic activity, measured by mitochondrial function in frozen/thawed samples, has been positively correlated with *in vivo *fertility in stallions [10] and bulls [11].

Sperm motility is essential for normal fertilization, and it is currently the most common parameter of "sperm quality", acting as an indirect measure of metabolic activity and sperm viability. Sperm motility after thawing and washing provided the most significant information for predicting donor sperm fertility potential, compared with fresh and thawed specimens used for insemination without any previous preparation protocol [12]. In addition studies showed that very low sperm motility was a good predictor of poor fertilization in IVF or ICSI [13,14]. Computer-assisted semen analysis (CASA) provides objective and reproducible data on a number of sperm motion parameters and it should enhance the value of motility assessment to fertility prognosis. In recent years there has been an increase in the use of these systems to evaluate semen quality [15-17] resulting in high correlations between several CASA motility parameters and the *in vivo *fertility of sperm from different species [in horses: [18]; in boar: [16]; in bulls: [19]].

Among other sperm tests, evaluation of DNA integrity has been considered important as early embryo development depends on the presence of normal DNA. After cryopreservation spermatozoa are particularly susceptible to DNA damage since freezing and thawing procedures lead to significant reduction in the level of spermatozoa antioxidant [20]. Therefore the assessment of DNA integrity is of high value in determining frozen/thawed semen quality. Significant relationship has been recorded between this parameter and fertility for bull frozen-thawed semen used for conventional AI [21,22].

Considering that combining different sperm function tests allows more accurate prediction of fertility (18), the aim of the present study was to investigate which parameters among a battery of analyses could predict subsequent spermatozoa *in vitro *fertilization ability and hence blastocyst output. To carry out our experiments we used Sarda goats as a model, since semen freezing and thawing procedures are well established for this species [23].

## Methods

### Chemicals

All chemicals in this study were purchased from Sigma Chemical CO. (St. Louis, MO, USA) unless stated otherwise.

### Animals and semen collection

All experimental procedures were carried out during goat breeding season (October - November) at the experimental facilities of the Department of Animal Biology at the University of Sassari, Italy (latitude 40°43' N). These facilities meet the requirements of the European Union for Scientific Procedure Establishments. This study followed ethical guidelines for care and use of agricultural animals for research. Ejaculates were obtained by artificial vagina from three adult Sarda male goats aged 2 years, maintained in an outdoor environment and fed a live-weight maintenance ration. Bucks were kept isolated in separated pens, but with visual contact between each others. Semen collection was repeated every 10 days, and 5 samples from each male were utilized for this study. Samples from each male were kept separated throughout all experimental procedures to highlight individual differences in sperm quality and freezability. Volume, concentration and total sperm output were recorded for each ejaculate collected. Volume was determined using a graduated micropipette, and concentration was evaluated by spectrophotometery using visible light after calculating a standard curve specific for this species. Total sperm output was calculated by multiplying this two parameters.

### Semen cryopreservation

Semen was diluted up to 400·10^6 ^sperm/mL in Tris-based extender (Tris 375 mM, citric acid 124 mM, glucose 41 mM) supplemented with egg yolk 20% (pH 7; osmolality 375 mOsm/kg) and glycerol (4%); it was cooled to 4°C over a period of 2 hr and equilibrated for 20 min before freezing. Finally, semen was frozen in pellet form (0.25 mL) on dry ice and then plunged into liquid nitrogen. Thawing was carried out by plunging a sterilized glass falcon tube containing the pellet in a 39°C water bath for 20 s.

The content of the Falcon tube was then emptied into a conical tube containing 3 mL of warmed synthetic oviduct fluid (SOF) supplemented with an antibiotic solution of streptomycin-penicillin (50 μg/mL - 50 IU/mL) and 0.1% (w/v) polyvinyl alcohol (PVA). Semen was centrifuged at 900·g for 3 min maintaining a constant temperature (35°C) to remove the freezing medium, the sperm pellet was then re-suspended in fresh medium and aliquots used for all the experimental procedures.

### Semen evaluation

The parameters analyzed included different semen molecular and cellular features, measured both before and after thawing, such as viability, motility parameters, ATP intracellular levels, and DNA integrity, which was evaluated only after thawing. An IVF test was performed to assess cryopreserved semen fertilizing ability.

#### Viability and motility parameters assessment

*In vitro *viability was assessed both before and after thawing by eosin-nigrosin stain [24].

Sperm motility parameters were assessed using a computer-assisted sperm analysis (CASA) system (Sperm Class Analyzer, S.C.A. v 3.2.0, Microptic S.L., Barcelona, Spain) with setting of 25 frames acquired to avoid sperm track overlapping, minimum contrast 10, minimum velocity of average path 30 μm/s, progressive motility > 80% straightness. This system has a specific set-up for goat sperm evaluation. For each sample, 5 μL subsample of sperm suspension was loaded into a pre-warmed analysis chamber with a depth of 10 μm (Makler Counting chamber, Sefi-Medical Instruments ltd., Biosigma S.r.l., Italy) and a minimum of 500 sperm per subsample were analyzed in at least four different microscopic fields.

Sperm motility was assessed at 37°C at 40 × using a phase contrast microscope. The percentage of progressive motile (P motile), and non progressive motile (NP motile) sperm, average velocity values of sperm (%; rapid, medium, slow and static), average path velocity (VAP, mm/s; the average velocity of the smoothed cell path), curvilinear velocity (VCL, mm/s; the average velocity measured over the actual point to point track followed by the cell), straight-line velocity (VSL, mm/s; the average velocity measured in a straight line from the beginning to the end of the track), linearity index (LIN, %; the average value of the ratio VSL/VCL), straightness index (STR, %; the average value of the ratio VSL/VAP), amplitude of lateral head displacement (ALH, mm; the mean width of the head oscillation as the sperm swim), beat cross-frequency (BCF, Hz; the frequency of sperm head crossing the average path in either direction) and wobble (WOB = (VAP/VCL) × 100, %; a measure of the oscillation of the actual trajectory about its spatial average path) were evaluated.

#### Extraction and measurement of intracellular ATP

Determination of intracellular ATP concentration was performed by the enzymatic assay as described by Zinellu et al. [25]. Briefly, 50 μL of fresh semen and 150 μL of frozen semen, (approximately 1.5 *10^9 ^cells/mL) were washed twice with 0.1 mL of cold physiological solution. For the extraction of nucleotides, 0.1 mL of ice-cold 0.6 M perchloric acid were added to each Eppendorf tube containing spermatozoa and kept for 15 min; after the suspension was centrifuged in an Eppendorf Microfuge (3 minutes at 10000 rpm) and the supernatant was neutralized with 15 μL of 3.5 M K_2_CO_3 _[26]. After one successive centrifugation in a Microfuge (3 min at 10000 rpm), the supernatant was analyzed spectrophotometrically with enzymatic assay.

ATP levels were measured spectrophotometrically at 340 nm using NADH-linked enzyme-coupled assays. The enzymatic spectrophotometric ATP assay was carried out at 37°C with a Beckman DU-7 spectrophotometer, and performed using the coupling enzymes, glucose 6 phosphate dehydrogenase (G6PD) and hexokinase (HK). Addition of excess HK (2 μL from 2 mg/mL) and G6PD (2 μL from 1 mg/mL) in the presence of excess glucose (8 μL from 18 mg/mL) and nicotinamide adenine dinucleotide phosphate (NADP+) (8 μL from 20 mg/mL) to perchloric extract (25 μL) and to 400 μL of TRAP buffer (0.1 M, pH 7.6), the reaction begins and ATP was determined from the formation of NADPH.

#### DNA integrity assessment

DNA damage was assessed only in frozen/thawed spermatozoa by single-cell gel electrophoresis (comet assay). Analysis of the shape and length of "comet" tail, just like the DNA content in the tail, gives an assessment of DNA damage. The neutral comet assay allows the detection of double-strand breaks by subjecting lysed cell nuclei to an electrophoretic field at neutral pH [27], here performed according to the method described by Sakkas et al. [28], with slight modifications. Briefly, sperm suspension (30 μl) was diluted in low-melting-point agarose (80 μl; 1% w/v). A 100-μl mixture of sperm-agarose was immediately pipetted onto 1% w/v normal-melting-point agarose-coated slides. Slides were immersed in ice-cold lysing solution (2.5 M NaCl, 100 mM EDTA, 10 mM Tris, 1% Triton X, and 10 mM dithiothreitol [DTT]; pH = 10) for 1 h at 4°C. Slides were then immersed in lysing solution supplemented with proteinase K (10 μg/mL). Incubation was performed during 1 h at 37°C. After this step, slides were rinsed in PBS and then placed in a horizontal electrophoresis tank filled with freshly prepared electrophoresis neutral buffer (Tris-acetate-EDTA [TAE], pH 7.3). Electrophoresis was performed at 10 V and 6 mA for 20 min. Following electrophoresis, the slides were neutralized with Tris-HCl buffer (pH 7.5) for 5 min and then fixed in methanol.

Slides were stained with propidium iodide (PI), mounted with a coverslip and analyzed under an epifluorescence microscope. Digital comet images were captured with an Olympus microscope equipped with a CCD camera and Olympus CellF software. Fifty comets were measured per replicate sample (i.e., slide circle) using CASP software (Comet Assay Software Project 1.2.2). Scored parameters included tail DNA %, Olive tail moment, and comet length (μm). Tail DNA % is a measurement of the proportion of total DNA that is present in the tail. The Olive tail moment is a global comet parameter expressed as [(tail mean × head mean) × (% tail DNA/100)] and used to quantify DNA damage [29]. The comet length is defined as the distance in micrometers between the right and left edges of the comet.

#### In vitro fertilization test

Ovaries were recovered at the local slaughterhouse and placed in Dulbecco's PBS at a temperature between 25°C and 35°C. After washing in fresh medium, ovaries were sliced using a micro-blade and the follicle content was released in medium TCM199 (with Earle's salts and bicarbonate) supplemented with 25 mmol Hepes, penicillin and streptomycin and 0.1% (w/v) of polyvinyl alcohol (PVA). The cumulus-oocyte complexes (COCs) comprised of 4-10 layers of granulosa cells and oocytes with a uniform cytoplasm, homogenous distribution of lipid droplets in the cytoplasm and with an outer diameter of about 90 μm (mean) were selected for the experimental procedure. The selected COCs, after three washes in the same fresh medium, were in vitro matured in TCM199 supplemented with 10% estrous goat serum, 10 μL/mL of FSH/LH and 100 μm of cysteamine. COCs were put in groups of 30-35, in 600 μL of the maturation medium in a four-well Petri dish (Nunclon, Nalgene Nunc International, Denmark), layered with 300 μL mineral oil and cultured for 24 hr in 5% CO_2 _in air at 39°C.

After maturation, the COCs were partially stripped of the granulosa cells and fertilized in vitro at 39°C and 5% CO_2_, 5% O_2 _and 90% N_2 _atmosphere in four-well Petri dishes (Nunclon). Synthetic oviduct fluid (SOF) containing 3% bovine serum albumin (BSA-fraction V) supplemented with 25 mm Hepes (sperm-SOF) was used for sperm preparation. For IVF, SOF medium was supplemented with 10% estrous goat serum, 20 μg/mL heparin and 1 μg/mL hipotaurine (IVF-SOF). Percoll^® ^gradients were prepared as described by Rosenkrans et al. [30]. In brief, 100% Percoll^® ^solution was mixed with a 10 × salt solution (NaCl 2.889 g; KCl 0.238 g; KH2PO4 0.116 g; CaCl2 0.112 g; Hepes 0.163 g; 50 mL of milli-Q water) to form 90% Percoll^® ^solution. A 45% Percoll^® ^solution was prepared from this by addition of an equal volume of sperm-SOF. The gradient was formed by pipetting 1 mL of 90% Percoll^® ^solution into a 15 mL conical tube and then overlaying it with 1 mL of 45% Percoll^® ^solution.

Frozen-thawed semen was placed onto the top of the 45% layer and then centrifuged at 2400 rpm at room temperature for 15 min through the gradient. After removal of supernatant, the resulting pellet was transferred in a sterilized conical glass tube below 1 mL of warmed IVF-SOF and incubated at 39°C in a humidified atmosphere at 5% CO_2 _in air for 15 min. Swim-up derived motile spermatozoa were diluted in IVF-SOF at a 1 × 10^6 ^spermatozoa/mL final concentration and incubated for 45 min at 39°C under 5% CO_2_, 5% O_2 _and 90% N_2_. For IVF, spermatozoa were co-incubated under mineral oil in four wells Petri dishes with a mean of 30 matured oocytes/well in the same atmosphere condition.

After 26 hrs, presumptive zygotes were mechanically denuded of their cumulus cells and cultured in four-well Petri dishes containing SOF + essential and non-essential amino acids at oviduct concentration + 0.4% BSA under mineral oil in maximum humidified atmosphere with 5% CO_2_, 5% O_2_, 90% N_2 _to blastocyst stage. At 40 h post-fertilization, uncleaved oocytes were stained with 1% lacmoid in fixing solution to evaluate chromatin configuration [31]. Oocytes showing decondensing sperm chromatin or pronuclei were classified as fertilized and added to the number of cleaved oocyte to calculate fertilization rates. Culture dishes were observed daily starting from the sixth day of culture and evolution to blastocysts was recorded.

### Statistical analysis

Statistical analysis were performed using the statistical software program Statgraphic Centurion XV (version 15.2.06 for Windows; StatPoint Technologies Inc., Warrenton, VA, USA) and a probability of P = 0.05 was considered to be the minimum level of significance. Differences in individual ejaculate traits and sperm parameters per male were studied by performing an ANOVA analysis. Differences in cleavage rate and embryo output per male were evaluated by performing a χ^2 ^test. In order to assess the predictive value of viability, CASA parameters and ATP intracellular concentration before and after thawing and of DNA integrity after thawing on subsequent embryo output after an in vitro fertility test, a logistic regression analysis was used. The dependent variable Y embryo output was expressed as a percentage (embryo obtained from each male over the number of fertilized oocytes) and the independent variables X were the means of sperm parameters (viability, motility and ATP intracellular concentration before and after thawing and DNA integrity after thawing) calculated for each ejaculate.

## Results

Table 1 summarized ejaculate traits recorded in three adult bucks during the breeding season. Total sperm output, which reflects differences found in both volume and concentration, was significantly lower in B compared with A and C.

**Table 1:** Ejaculate traits recorded in three adult male goats (A, B and C) during the breeding season.

	**Volume** **(μL)**	**Concentration** **(sperm × 10^6^/mL)**	**Total sperm output** **(sperm × 10^6^)**
A	891.2 ± 567.3^a^	1787.06 ± 515.5^a^	1584.7 ± 1134.4^a^
B	461.3 ± 227.9^b^	1815.13 ± 801.4^a^	833.8 ± 474.5^b^
C	689.4 ± 370.8^a, b^	2373.9 ± 607.7^b^	1543.7 ± 725.8^a^

^a, b ^Within the same column: a ≠ b p < 0.05 (ANOVA one way).

As summarized in table 2, individual differences in semen parameters were evident only for semen viability after thawing. Freezing process significantly reduced semen viability in all the samples analyzed (p < 0.0001), but semen collected from A maintained a higher viability compared with B (p < 0.05), while C did not differ from the others.

**Table 2:** Analysis of several cellular and molecular parameters in fresh and frozen/thawed semen collected from three adult male goats (A, B and C).

		**A**		**B**		**C**	
		**Fresh**	**Frozen**	**Fresh**	**Frozen**	**Fresh**	**Frozen**
Viability (%)		80.4 ± 6.6	60.2 ± 7.9^α^	74.4 ± 9.1	48.9 ± 11^β^	75.5 ± 9.7	54.7 ± 11.2^α, β^
							
CASA motility parameters*	NP motile (%)	58.2 ± 4.9	58.2 ± 14.2	50.5 ± 6.9	57.8 ± 1.6	54 ± 12^a^	68 ± 8^b^
	P motile (%)	31.7 ± 5.3	25.7 ± 9.3	32.4 ± 10.7	19.6 ± 11.3	27.2 ± 10.6	20.4 ± 6
	Rapid (%)	65.8 ± 16.4	49.7 ± 15.8	60.8 ± 22.8	45.4 ± 12	56.4 ± 19.2	47.3 ± 4.5
	Medium (%)	12.4 ± 8.6	15.3 ± 5.6	9.6 ± 6.4	13.8 ± 5.3	13.4 ± 10.3	18 ± 3.7
	Slow (%)	11.8 ± 7.7^a^	18.9 ± 3.1^b^	12.7 ± 8.5^a^	23.8 ± 6.1^b^	13.2 ± 5.7^c^	22.8 ± 1.8^d^
	Static (%)	10 ± 5	16.1 ± 17	16.9 ± 12	17 ± 7	16.9 ± 13.4	11.9 ± 3
	VAP (μm/sec)	76.8 ± 23.6	57.9 ± 9.5	69.1 ± 28.9	49.8 ± 12.1	67 ± 23.6	50.5 ± 7.8
	VCL (μm/sec)	105.5 ± 23.4^a^	83.1 ± 12.1^b^	96 ± 27.5	74.2 ± 23	94.6 ± 22.3	76.3 ± 9.3
	VSL (μm/sec)	53.5 ± 17.4	41 ± 7.8	50.3 ± 23.5	34.9 ± 14.8	47 ± 18.1	34.2 ± 6.6
	LIN (%)	50.1 ± 9.4	49.7 ± 8.1	50.9 ± 13.7	46.2 ± 8.6	48.7 ± 10.2	44.6 ± 5.6
	STR (%)	69.4 ± 5.4	70.9 ± 6.1	71.5 ± 6.7	69 ± 5.7	69.6 ± 7.3	67.3 ± 4.4
	WOB (%)	71.8 ± 10	69.8 ± 6.1	70.4 ± 14.2	66.5 ± 6.5	69.5 ± 9.6	66.1 ± 4.8
	ALH (μm)	3.3 ± 0.5	3.4 ± 0.5	3.2 ± 0.7	3.6 ± 0.4	3.8 ± 0.6	3.5 ± 0.4
	BCF (Hz)	8.7 ± 1.3	8.2 ± 0.8	9.6 ± 1.9	7.9 ± 1.3	8.9 ± 1.9	7.9 ± 1.4
							
ATP concentration (nmolATP/10^9 ^spermatozoa)		107.4 ± 50.61	48 ± 37	66.5 ± 28.9	27.5 ± 29.5	147.8 ± 55.5^a^	62.7 ± 45.4^b^

*P motile: progressive motile sperm; NP motile: non progressive motile sperm; rapid, medium, slow and static: average velocity values of sperm (%); VAP: average path velocity (mm/s); VCL: curvilinear velocity (mm/s); VSL: straight-line velocity (mm/s); LIN: linearity index (%);STR: straightness index (%); ALH: amplitude of lateral head displacement (mm); BCF: beat cross-frequency (Hz); WOB: wobble (%).

^α, β ^Within the same row but among different groups different superscripts indicate statistical difference (ANOVA one way): α ≠ β p < 0.05.

a, b, c, d, Within the same row and group different superscripts indicate statistical difference (ANOVA one way): a ≠ b p < 0.05; c ≠ d p < 0.01.

The analysis of CASA parameters did not reveal any difference among the 3 individuals in the sperm motility pattern showed both before and after thawing. The percentage of non progressive motile spermatozoa increased after thawing in C, while no differences were recorded between fresh and frozen semen in A and B. In the same way, VCL increased after thawing in A, while no differences were recorded between fresh and frozen semen in the others.

ATP intracellular concentration showed a wide range of variability both in fresh and frozen samples. Considering inter-individual variations, results approached significance in fresh samples (p = 0.0518); in particular, semen collected from B showed lower ATP concentration compared with C, while A did not differ from the others. Freezing and thawing significantly reduced ATP intracellular concentration in samples collected from C (p < 0.05), while no difference between fresh and frozen samples were detected in A and B. Furthermore, no difference was found between the three individuals in ATP intracellular concentration after thawing.

DNA integrity, as evaluated by the comet assay, was assessed only in frozen semen (table 3). While no difference was found among the three individuals in percentage tail DNA, olive tail moment was lower in B than in A and C (p < 0.01). In addition, comet length was significantly higher in C than in A and B (p < 0.01).

**Table 3:** Differences in comet parameters in frozen/thawed spermatozoa collected from three adult male goats (A, B and C).

	**Tail DNA %**	**Olive tail moment**	**Comet lenght (μm)**
A	13.6 ± 1.9	6 ± 0.9^a^	99.7 ± 1.5^a^
B	10.2 ± 1.8	1.9 ± 0.8^b^	99.3 ± 1.5^a^
C	14.9 ± 1.8	5.2 ± 0.9^a^	110.9 ± 1.5^b^

^a, b ^Within the same column different superscripts indicate statistical difference: (ANOVA one way) a ≠ b p < 0.01.

Data are expressed as mean ± SE.

Result of the *in vitro *fertility test are summarized in table 4. Spermatozoa collected from A and B lead to higher cleavage rates (p < 0.01) and blastocysts output (p < 0.05) compared with C (table 4). In addition, a faster embryo developmental kinetic was observed when oocytes were fertilized with spermatozoa collected from A and B compared with C, as revealed by the higher number of expanded blastocyst obtained on day 7 post-fertilization.

**Table 4:** Differences in *in vitro *cleavage rates and blastocyst output obtained after in vitro fertilization of goat oocytes with cryopreserved semen obtained from three adult male goats (A, B and C).

**Male**	**n**	**Oocyte Fertilization (%)**	**Cleavage (%)**	**Expanded blastocyst (%)**			
				**7 dpf**	**8 dpf**	**9 dpf**	**total**
A	460	362 (78.7)	226 (62.4)^a^	10 (4.4)^a^	19 (8.4)^c^	13 (5.7)^c^	42 (37.2)^a^
B	440	358 (81.4)	194 (54.2)^b^	7 (3.6)^a^	17 (8.8)^c^	8 (4.1)^c, d^	32 (33)^a^
C	440	352 (80.0)	176 (50.0)^b^	1 (0.5)^b^	7 (4.0)^d^	4 (2.3)^d^	12 (13.6)^b^

Dpf = days post-fertilization

^a, b, c, d ^Within the same column: Chi square test: a ≠ b p < 0.01; c ≠ d p < 0.05.

Logistic regression analysis, after a stepwise forward selection of the factors, reported one model with high percentage of concordance between a set of the considered variables and in vitro embryo output. The model explained a deviance of 72% (p < 0.0001), directly related with the mean percentage of rapid sperm in fresh semen (p < 0.01), semen viability after thawing (p < 0.01), and with two of the three comet parameters considered, i.e tail DNA percentage (p < 0.0001) and comet length (p < 0.0001). ATP levels neither before nor after thawing could be related to subsequent in vitro embryo output. On the other hand, it should be pointed out that considering tail DNA percentage and comet length alone the model could explain a deviance of 57%.

## Discussion

This study aimed to investigate which parameters among a battery of analyses could predict frozen/thawed spermatozoa fertility, as evaluated by the IVF test, in a goat model. *In vitro *fertilization has been reported to be one of the most adequate parameters for semen fertility prediction since it evaluates the spermatozoa-oocyte interactions occurring during in vivo fertilization, allowing measurement of different endpoints in the early stages of the embryo development [2]. In addition, in vitro fertilization assays provide the advantage of being able to culture subsequent zygotes, providing information on the genetic normalcy of the sperm [32]. Semen from bulls, boars and rams has been tested and statistically significant relationship between IVF fertility and fertility *in vivo *was found both in retrospective studies [33,34] and prospectively [35].

Three main conclusion can be drawn from the current study; first, DNA integrity represents a factor able to strongly influence in vitro embryo outcome with frozen/thawed semen; second, the combination of a few sperm function parameters, such as percentage of rapid spermatozoa in fresh semen, viability after thawing and comet parameters (tail DNA percentage and comet length) allows an accurate prediction of subsequent in vitro embryo output; and third sperm energetic status, as evaluated by ATP intracellular levels, cannot be related to ART outcome.

Considerable effort is being invested in identifying markers for functional sperm capacity that can more accurately predict fertility. DNA integrity represents an important parameter in the evaluation of frozen/thawed semen functionality. Although DNA repair occurs in developing sperm [36], it is terminated as transcription and translation cease post-spermiogenesis [37]. As a result, sperm have no mechanism to repair DNA damage incurred during their transit and storage in the epididymis, post-ejaculation or during freezing procedures. In particular, freezing seems to affect chromatin structure [38,39], and DNA damage from cryopreservation in semen from infertile men has been detected using the comet assay [40].

In the present study, considering that individual differences emerged among the three donors in the degree of DNA damage after thawing, we found a high predictive value of comet parameters in frozen/thawed semen on subsequent in vitro embryo output. IVF experiments with gamma irradiated sperm showed that sperm with severe DNA damage remained functionally intact at the level of membrane and organelle and motility parameters [41]. In fact, the DNA damaged sperm showed normal zona pellucida binding characteristics and even the fertilization and cleavage rates of the fertilized oocytes remained normal. However, about all four to eight cell embryo stages initiated apoptosis [41,42]. Thus, the reproductive failure, caused by DNA aberrations, appears not at the level of fertilization but at the onset of embryonic DNA expression, when the paternal genome exerts a major influence [43]. Thus, in assisted conception cycles in humans, preimplantation development is negatively correlated with DNA damage assessed by a variety of methods including in situ-nick translation [44], comet assay [45], TUNEL [46,47] and sperm chromatin structure assay (SCSA) [48,49]. In IVF procedures a negative correlation with fertilization, kinetic of embryo development and implantation rate were correlated with higher sperm DNA fragmentation in sperm samples [50]. Other authors failed to find a predictive value on the outcome of intra-uterine insemination (IUI), IVF and intracytoplasmatic sperm injection (ICSI) of spermatozoa DNA integrity, as evaluated by the SCSA when measured after density gradient centrifugation [51], and concluded on the basis of previous study [52], that the prognostic ability of SCSA is limited to analyses performed on neat semen.

In the current study we assessed DNA integrity after freezing and thawing and found a high prognostic value of this parameter on subsequent in vitro embryo output. In addition, the deviance explained by the logistic regression model rose from 57% to 72% when other two factor were included, i.e percentage of rapid spermatozoa in fresh semen and semen viability after thawing.

Semen viability after thawing reflects the ability of spermatozoa to withstand freezing and thawing procedures, i.e in some terms sperm freezability. It is well known that this parameter is highly subjective and likely to be related to genetic differences [53]. On the other hand, CASA parameters evaluated both before and after thawing were excluded by the model, a part from percentage of rapid spermatozoa in fresh semen. This parameter may reflect overall ejaculate quality.

There have been many reports that poor motility is associated with low IVF fertilization rates [e.g. [54-56]]. For many years, the assessment of motility has been regarded as one of the most informative aspects of semen analysis [57].

Following the introduction of CASA, a wide variety of parameters have been found to be associated with fertilization rates in vitro. Those selected as the best predictors include: linearity in semen and percentage spermatozoa in insemination medium with velocities between 10 and 20 μm/sec [58], % progressively motile in semen or ALH and VAP in medium (zona free hamster oocytes; [59]), % spermatozoa with rapid motility; % sperm motile and VCL all in prepared spermatozoa [56]; VCL [55]; and % of progressive motile spermatozoa (hamster oocyte test; [60]). It should be considered that many of the sperm motility measurements derived from CASA analysis are significantly correlated with each other [58] and there are significant variations in IVF and measurement techniques between laboratories. It is not surprising that whereas it is almost universally acknowledged that sperm motility is an important factor in predicting IVF outcome, the parameter taken as the best index should vary between studies.

In the present study we did not found any predictive value of spermatozoa energetic status, as evaluated by ATP intracellular concentration before and after thawing, on IVF outcome. In other studies, sperm oxygen consumption has been correlated with bull fertility and measurement of total ATP formation has been proposed as a test for bull fertilizing ability after freezing and thawing [8]. We reported for Griffon vulture spermatozoa that an higher ATP intracellular concentration in fresh semen was followed by a longer survival in vitro after cryopreservation [9]. Furthermore, ATP values correlated positively with sperm viability both before and after cryopreservation [9]. It can be speculated that this parameter may provide useful information if measured at different time points after thawing since it could reflects semen ability to restore ATP intracellular level after the severe energetic loss caused by cryopreservation.

In conclusion, the model proposed here represent one of the many possible ways to explain differences found in embryo output following IVF with different goat semen donors. In our system, DNA integrity, as evaluated by a high sensible method such as the comet assay, has a high predictive value on IVF outcome with frozen/thawed semen (deviance explained: 57%). Besides, adding to the model other two factors, such as of rapid spermatozoa in fresh semen and viability after thawing a 72% of deviance in embryo out put could be explained. This model may represent a useful tool to select the most suitable donors for semen cryopreservation.

## Competing interests

To the authors' best knowledge, no competing interests of any nature arise from the current publication.

## Authors' contributions

FB participated in the design of the study, carried out experimental analysis, performed the statistical analysis and drafted the manuscript. MM performed the semen collection and analyses, and helped drafting the manuscript. VP performed the comet assays and the ATP analyses. SS, AS and VS helped in sample collection and in drafting of the manuscript and participated in the design of the study. PM helped performing the comet assays and the ATP analyses. GGL participated in the design of the study and performed the statistical analysis. SN conceived the study, participated in its design and coordination and helped to draft the manuscript. All co-authors provided inputs during final manuscript preparation. All authors read and approved the final manuscript.
